# Modelling the dynamics of Pine Wilt Disease with asymptomatic carriers and optimal control

**DOI:** 10.1038/s41598-020-67090-7

**Published:** 2020-07-10

**Authors:** Muhammad Altaf Khan, L. Ahmed, Prashanta Kumar Mandal, Robert Smith, Mainul Haque

**Affiliations:** 1 Informetrics Research Group, Ton Duc Thang University, Ho Chi Minh City, Vietnam; 2 Faculty of Mathematics and Statistics, Ton Duc Thang University, Ho Chi Minh City, Vietnam; 3 Department of Mathematics, City University of Science and Information Technology, Peshawar, Pakistan; 4 Department of Mathematics, Visva-Bharati University, Santiniketan, 731 235 W.B. India; 5 Department of Mathematics and Faculty of Medicine, The University of Ottawa, Ottawa, ON K1N 6N5 Canada; 6 Department of Mathematics and Physics University of Portsmouth, Portsmouth, PO1 2UP UK

**Keywords:** Computational models, Environmental impact, Environmental impact, Applied mathematics, Applied mathematics

## Abstract

Pine wilt disease is a lethal tree disease caused by nematodes carried by pine sawyer beetles. Once affected, the trees are destroyed within a few months, resulting in significant environmental and economic losses. The role of asymptomatic carrier trees in the disease dynamics remains unclear. We developed a mathematical model to investigate the effect of asymptomatic carriers on the long-term outcome of the disease. We performed a stability and sensitivity analysis to identify key parameters and used optimal control to examine several intervention options. Our model shows that, with the application of suitable controls, the disease can be eliminated in the vector population and all tree populations except for asymptomatic carriers. Of the possible controls (tree injection, elimination of infected trees, insecticide spraying), we determined that elimination of infected trees is crucial. However, if the costs of insecticide spraying increase, it can be supplemented (although not replaced entirely) by tree injection, so long as some spraying is still undertaken.

## Introduction

Among the vector-borne diseases of trees and plants, the most destructive are a red ring disease of palms and Pine Wilt Disease (PWD), whose causative agent is pine wood nematodes (PWNs)^1^. The vector for PWD is the pine sawyer beetle, which transfers nematodes to healthy host pine trees and usually kills host trees within a few months of infection. The lack of resin exudation of bark wounds become visible as a first symptom. The foliage become pale green in the second stage, yellow in the third stage and finally become reddish brown when the trees fail to resist against the disease. It is well-established that PWD has three different transmission paths: the first happens during maturation feeding^2^; the second during oviposition of the mature female on recently cut, dying, or dead pine trees through the oviposition wounds^3^; and the third is horizontal transmission, which happens during mating^4^.

A number of epidemiological studies have been carried out to investigate the transmission dynamics of pine wilt disease^5–8^. These models investigating the spread and control of PWD are used to describe the host–vector interaction between nematode-carrying pine sawyers and pine trees. Lee^9^ presented an epidemiological model of PWD and developed optimal-control strategies for the prevention of PWD. Khan *et al*.^10^ introduced a dynamical model of PWD and investigates the stability of the disease with saturated incidence rate. They classified the total host tree size into three states: susceptible, exposed and infected host pine trees, while the vector size was also classified into three similar states. Ozair^11^ included horizontal transmission and nonlinear incidence. The global stability of PWD in a model with nonlinear incidence rates was analyzed by Lee^5^. Optimal control has been used to study a variety of infectious disease^12–16^, including plant diseases^17–19^.

Asymptomatic carrier cases can play a critical role in the subsequent spread of PWD^20^. Asymptomatic infection increases the density of infected vectors, which further increases the level of infection in the host. Studies on asymptomatic infection in pine trees show that asymptomatic infected trees may remained infected for up to a year and may ultimately die^21^. Mathematical models that address pine tree dynamics with asymptomatic infections have previously been considered^22,23^. The effect of asymptomatic infection on neighboring trees has also been studied^20^.

Here, we develop a dynamic model of PWD incorporating an asymptomatic carrier class and examine control policies that minimize implementation costs while protecting forests from the disease. To the best of our knowledge, none of the previous mathematical studies used optimal control to explore the transmission dynamics of the PWD in the presence of the asymptomatic carriers.

## Model formulation

The total host (pine wood trees) and vector (beetles) are represented by *N*_*H*_(*t*) and *N*_*V*_(*t*), respectively. *N*_*H*_(*t*) is further classified into four epidemiological classes: susceptible pine trees *S*_*H*_(*t*), exposed pine trees *E*_*H*_(*t*), asymptomatic carrier pine trees *A*_*H*_(*t*) and infected pine trees *I*_*H*_(*t*). *N*_*V*_(*t*) is classified into three epidemiological classes: susceptible beetles *S*_*V*_(*t*), exposed beetles *E*_*V*_(*t*) and infected beetles *I*_*V*_(*t*).

**Figure 1:**
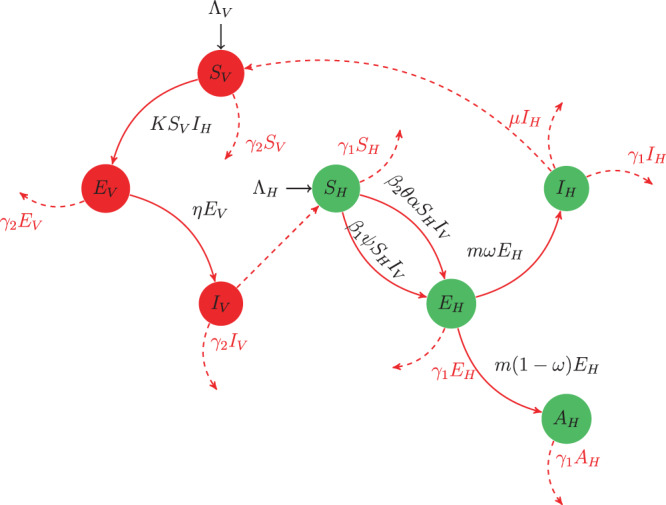
Flow chart for the transmission of PWD. The short dashed arrows indicate the natural and the disease-specific death rates in each compartment. The long dashed arrows represent the interaction between the vector and pine trees. The long solid arrows represent the transition between compartments due to disease. The short solid arrows represent the recruitment.

The recruitment rates of host trees and beetles are represented, respectively, by Λ_*H*_ and Λ_*V*_, while the natural death rates of host pine trees and vector beetles are denoted by *γ*_1_ and *γ*_2_, and the disease mortality rate of host pine trees is represented by *μ*. Here, *m* and *η* are the respective rates of progression from the exposed class to the infected class in the host and vector populations. The term *β*_1_*ψS*_*H*_*I*_*V*_ denotes the incidence rate, where *β*_1_ is the rate of transmission and *ψ* is the average number of daily contacts with vector adult beetles during maturation. *β*_2_ is the rate at which an infected beetle transmits a nematode through oviposition, with the average number of oviposition contacts per day denoted by *θ*. The termination of oleoresin exudation in susceptible trees without infection of nematode is denoted by *α*. We thus interpret *β*_2_*θα* as the transmission through oviposition, and hence *β*_2_*θαS*_*H*_*I*_*V*_ represents the number of new infections. A fraction *ω* (0 ≤ *ω* ≤ 1) of the exposed tree class generates symptomatic infection, while the remaining fraction (1 − *ω*) generates asymptomatic infection. The vector incidence rate is given by the term *KI*_*H*_*S*_*V*_^15^. The schematic diagram for the PWD model is shown in Fig. 1.

The model is thus given by

1 $$\begin{array}{rcl}{S{\prime} }_{H} & = & {\Lambda }_{H}-{\beta }_{1}\psi {S}_{H}{I}_{V}-{\beta }_{2}\theta \alpha {S}_{H}{I}_{V}-{\gamma }_{1}{S}_{H},\\ {E{\prime} }_{H} & = & {\beta }_{1}\psi {S}_{H}{I}_{V}+{\beta }_{2}\theta \alpha {S}_{H}{I}_{V}-({\gamma }_{1}+m){E}_{H},\\ {A{\prime} }_{H} & = & m(1-\omega ){E}_{H}-{\gamma }_{1}{A}_{H},\\ {I{\prime} }_{H} & = & m\omega {E}_{H}-({\gamma }_{1}+\mu ){I}_{H},\\ {S{\prime} }_{V} & = & {\Lambda }_{V}-K{S}_{V}{I}_{H}-{\gamma }_{2}{S}_{V},\\ {E{\prime} }_{V} & = & K{S}_{V}{I}_{H}-({\gamma }_{2}+\eta ){E}_{V},\\ {I{\prime} }_{V} & = & \eta {E}_{V}-{\gamma }_{2}{I}_{V},\end{array}$$

with initial conditions

$${S}_{H}(0)\ge 0,{E}_{H}(0)\ge 0,{A}_{H}(0)\ge 0,{I}_{H}(0)\ge 0,{S}_{V}(0)\ge 0,{E}_{V}(0)\ge 0,{I}_{V}(0)\ge 0.$$

The total population sizes of host and vector are given by

$${N}_{H}(t)={S}_{H}(t)+{E}_{H}(t)+{A}_{H}(t)+{I}_{H}(t),{N}_{V}(t)={S}_{V}(t)+{E}_{V}(t)+{I}_{V}(t).$$

For biological realism, we study the behaviour of the system (1) in the closed set

$$\begin{array}{rcl}\Psi  & = & \{({S}_{H},{E}_{H},{A}_{H},{I}_{H},{S}_{V},{E}_{V},{I}_{V})\in {{\mathbb{R}}}_{+}^{7}|0\le {S}_{H}+{E}_{H}+{A}_{H}+{I}_{H}\le \frac{{\Lambda }_{H}}{{\gamma }_{1}},\\  &  & 0\le {S}_{V}+{E}_{V}+{I}_{V}\le \frac{{\Lambda }_{V}}{{\gamma }_{2}}\}.\end{array}$$

Nonnegative solutions of system (1) can be easily verified for appropriate initial values. The first four equations of (1) imply that

$$\frac{d({S}_{H}+{E}_{H}+{A}_{H}+{I}_{H})}{dt}\le {\Lambda }_{H}-{\gamma }_{1}({S}_{H}+{E}_{H}+{A}_{H}+{I}_{H}).$$

By comparison theorem presented in^24^, there exists *t*_1_ > 0, such that

$${S}_{H}+{E}_{H}+{A}_{H}+{I}_{H}\le \frac{{\Lambda }_{H}}{{\gamma }_{1}}\equiv {N}_{1}\,{\rm{for}}\,t > {t}_{1}.$$

Similarly, adding the last three equations of the system (1), we get

$$\frac{d({S}_{V}+{E}_{V}+{I}_{V})}{dt}={\Lambda }_{V}-{\gamma }_{2}({S}_{V}+{E}_{V}+{I}_{V}).$$

Using the comparison theorem again, there exists *t*_2_ > *t*_1_, such that

$${S}_{V}+{E}_{V}+{I}_{V}\le \frac{{\Lambda }_{V}}{{\gamma }_{2}}\equiv {N}_{2}\,{\rm{for}}\,t > {t}_{2}.$$

Hence, the solutions of the system (1) are bounded.

In the Supplementary Material, we determine ${ {\mathcal R} }_{0}$ and prove that the disease-free equilibrium (DFE) is globally asymptotically stable, which also rules out the possibility of a backward bifurcation. We also show that the endemic equilibrium is globally asymptotically stable, under certain conditions.

## Sensitivity analysis of threshold dynamic

Due to uncertainties in experimental data, determining accurate outcomes from an epidemiological system is difficult^25^. To compensate for these uncertainties, we use partial rank correlation coefficients (PRCCs) to identify the impact of all parameters on ${ {\mathcal R} }_{0}$. This technique measures the degree of the relationship between inputs and output of the system. Positive PRCCs indicate parameters that increase ${ {\mathcal R} }_{0}$ when they are increased, while negative PRCCs indicate parameters that decrease ${ {\mathcal R} }_{0}$ when they are increased. Parameters with PRCCs values greater than 0.4 in magnitude have a significant effect on the outcome.

**Figure 2:**
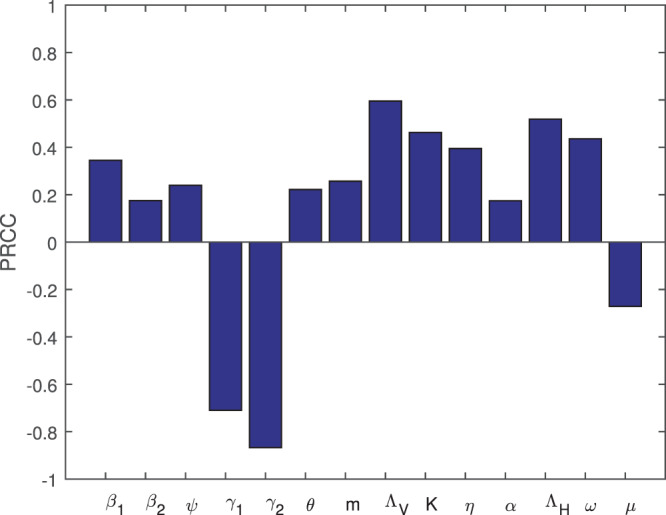
Sensitivity of *R*_0_ to all input parameters.

Figure 2 illustrates the effect of parameter variations on ${ {\mathcal R} }_{0}$ for all fourteen parameters. Clearly, ${ {\mathcal R} }_{0}$ is most sensitive to *γ*_1_ and *γ*_2_, the natural death rates of pine trees and beetles, respectively; the latter can be controlled using insecticide (*u*_3_), while the former can be partially controlled by eliminating infected trees (*u*_2_). ${ {\mathcal R} }_{0}$ is also sensitive to the birth rates of pine trees and beetles, the latter of which can be controlled using insecticide (*u*_3_). The transmission rate *K* is also a sensitive parameter, which can be controlled by nematicide-injection and vaccination (*u*_1_).

## Optimal control strategies

In this section, we introduce *u*_1_, *u*_2_ and *u*_3_ as three control measures that can affect PWD. The force of infection in the pine-tree population is reduced by (1 − *u*_1_), where precautionary measures efforts are denoted by *u*_1_; for example nematicide injection and vaccination. To keep the host tree population safe and to prevent infection, the nematicide-injection preventative control measure is used. We use the control variable *u*_2_ to describe elimination of infected host trees. Supplementary infections are extremely reduced by demolition and elimination of infected host trees. The removal of these infected trees guarantees that eggs, larvae and pupa that are occupying the host pines are devasted. Our third control variable represents spraying of insecticide and larvacide to kill adult insects and reduce the vector birth rate.

Model (1) is modified for optimal control as follows:

2 $$\begin{array}{ccc}{S{\rm{{\prime} }}}_{H} & = & {\Lambda }_{H}+c{N}_{H}-{\beta }_{1}\psi {S}_{H}{I}_{V}(1-{u}_{1})-{\beta }_{2}\theta \alpha {S}_{H}{I}_{V}(1-{u}_{1})-{\gamma }_{1}{S}_{H},\\ {E{\rm{{\prime} }}}_{H} & = & {\beta }_{1}\psi {S}_{H}{I}_{V}(1-{u}_{1})+{\beta }_{2}\theta \alpha {S}_{H}{I}_{V}(1-{u}_{1})-({\gamma }_{1}+m){E}_{H},\\ {A{\rm{{\prime} }}}_{H} & = & m(1-\omega ){E}_{H}-{\gamma }_{1}{A}_{H},\\ {I{\rm{{\prime} }}}_{H} & = & m\omega {E}_{H}-({\gamma }_{1}+\mu ){I}_{H}-{u}_{2}{b}_{1}{I}_{H},\\ {S{\rm{{\prime} }}}_{V} & = & {\Lambda }_{V}(1-{u}_{3})-K{S}_{V}{I}_{H}(1-{u}_{1})-{\gamma }_{2}{S}_{V}-{b}_{0}{u}_{3}{S}_{V},\\ {E{\rm{{\prime} }}}_{V} & = & K{S}_{V}{I}_{H}(1-{u}_{1})-({\gamma }_{2}+\eta ){E}_{V}-{b}_{0}{u}_{3}{E}_{V},\\ {I{\rm{{\prime} }}}_{V} & = & \eta {E}_{V}-{\gamma }_{2}{I}_{V}-{b}_{0}{u}_{3}{I}_{V},\end{array}$$

with nonnegative initial conditions. The control functions *u*(*t*) = (*u*_1_, *u*_2_, *u*_3_) ∈ *U* associated to the variables *S*_*H*_, *E*_*H*_, *A*_*H*_, *I*_*H*_, *S*_*V*_, *E*_*V*_ and *I*_*V*_ satisfy

3 $$U(t)=\{({u}_{1},{u}_{2},{u}_{3})\,are\,Lebesgue\,measurable,0\le {u}_{i}\le 1,t\in [0,T],i=1,2,3\}.$$

The constants *b*_0_ and *b*_1_ are removal-rate constants whose inverses correspond to the average time spent in the relevant compartment. Since it is unlikely that infected trees will be removed within one day of infection, we set *b*_1_ = 1; hence the range 0 ≤ *u*_2_ ≤ 1 corresponds to a removal time between 1 day and infinite time. The objective functional for the optimal-control problem is

4 $$\begin{array}{ccc}J({u}_{1},{u}_{2},{u}_{2}) & = & {\int }_{0}^{T}\,[{L}_{1}{E}_{H}+{L}_{2}{A}_{H}+{L}_{3}{I}_{H}+{L}_{4}{N}_{V}\\  &  & +\frac{1}{2}({B}_{1}{u}_{1}^{2}+{B}_{2}{u}_{2}^{2}+{B}_{3}{u}_{3}^{2})]dt,\end{array}$$

subject to the control system (2). The constants *L*_1_, *L*_2_, *L*_3_, *L*_4_, *B*_1_, *B*_2_ and *B*_3_ are the weight or balancing constants, which measure the relative cost of interventions over the interval [0, *T*]. We seek optimal controls ${u}_{1}^{\ast },{u}_{2}^{\ast },{u}_{3}^{\ast }$, such that

5 $$J({u}_{1}^{\ast },{u}_{2}^{\ast },{u}_{3}^{\ast })=\mathop{{\rm{\min }}}\limits_{U}\{{u}_{1},{u}_{2},{u}_{3}\}.$$

Clearly, the equations in the control system (2) are bounded above, and thus we can apply the results in^26^ to model (2). Moreover, the set of control variables and the state variables is nonempty, and the set of control variables denoted by *U* is closed and convex. In the control problem (2), the right-hand side is continuous and bounded above by state variables and a sum of the bounded control, and can be expressed as a linear function of *U* having state- and time-dependent coefficients. Hence there exists constants *m* > 1 and *l*_1_, *l*_2_ > 0 such that the integrand *L*(*y*, *u*, *t*) of the objective functional *J* is convex and satisfies

$$L(y,u,t)\ge {l}_{1}{(|{u}_{1}{|}^{2}+|{u}_{2}{|}^{2}+|{u}_{3}{|}^{2})}^{\frac{m}{2}}-{l}_{2}.$$

We apply the results presented in^27^ to justify the existence of (2) and to obey the above conditions. Clearly, the set of control and state variables are bounded and nonempty. The solutions are bounded and convex. Therefore the system is bilinear in the control variables. We verify the last condition:

$$\begin{array}{c}{L}_{1}{E}_{H}+{L}_{2}{A}_{H}+{L}_{3}{I}_{H}+{L}_{4}{N}_{V}+\frac{1}{2}({B}_{1}{u}_{1}^{2}+{B}_{2}{u}_{2}^{2}+{B}_{3}{u}_{3}^{2})\\ \,\ge \,{l}_{1}{(|{u}_{1}{|}^{2}+|{u}_{2}{|}^{2}+|{u}_{3}{|}^{2})}^{\frac{m}{2}}-{l}_{2},\end{array}$$

where *L*_1_, *L*_2_, *L*_3_, *L*_4_, *B*_1_, *B*_2_, *B*_3_, *l*_1_, *l*_2_ > 0 and *m* > 1. We have thus proved the following theorem.

**Theorem 1.** *For the objective functional* (4) *and the control set* (3) *subject to the control system* (2), *there exists an optimal control* ${u}^{\ast }=({u}_{1}^{\ast },{u}_{2}^{\ast },{u}_{3}^{\ast })$ *such that* $({u}_{1}^{\ast },{u}_{2}^{\ast },{u}_{3}^{\ast })={\min }_{{\rm{U}}}J({u}_{1},{u}_{2},{u}_{3})$.

In order to get the solution of the control problem, it is necessary to obtain the Lagrangian and the Hamiltonian of (2). The Lagrangian *L* is expressed as

$$\begin{array}{ccc}L({E}_{H},{A}_{H},{I}_{H},{N}_{V},{u}_{1},{u}_{2},{u}_{3}) & = & {L}_{1}{E}_{H}+{L}_{2}{A}_{H}+{L}_{3}{I}_{H}+{L}_{4}{N}_{V}\\  &  & +\,\frac{1}{2}({B}_{1}{u}_{1}^{2}+{B}_{2}{u}_{2}^{2}+{B}_{3}{u}_{3}^{2}).\end{array}$$

By choosing *X* = (*S*_*H*_, *E*_*H*_, *I*_*H*_, *S*_*H*_, *E*_*H*_, *I*_*H*_), *U* = (*u*_1_, *u*_2_, *u*_3_) and *λ* = (*λ*_1_, *λ*_2_, *λ*_3_, *λ*_4_, *λ*_5_, *λ*_6_, *λ*_7_), the Hamiltonian can be written

6 $$\begin{array}{rcl}H(X,U,\lambda ) & = & L({E}_{H},{A}_{H},{I}_{H},{N}_{V},{u}_{1},{u}_{2},{u}_{3})+{\lambda }_{1}[{\Lambda }_{H}+c{N}_{H}-{\beta }_{1}\psi {S}_{H}{I}_{V}(1-{u}_{1})\\  &  & -\,{\beta }_{2}\theta \alpha {S}_{H}{I}_{V}(1-{u}_{1})-{\gamma }_{1}{S}_{H}]+{\lambda }_{2}[{\beta }_{1}\psi {S}_{H}{I}_{V}(1-{u}_{1})+{\beta }_{2}\theta \alpha {S}_{H}{I}_{V}(1-{u}_{1})\\  &  & -\,({\gamma }_{1}+m){E}_{H}]+{\lambda }_{3}[m(1-\omega ){E}_{H}-{\gamma }_{1}{A}_{H}]+{\lambda }_{4}[m\omega {E}_{H}-({\gamma }_{1}+\mu ){I}_{H}-{u}_{2}{I}_{H}]\\  &  & +\,{\lambda }_{5}[{\Lambda }_{V}{N}_{V}(1-{u}_{3})-K{S}_{V}{I}_{H}(1-{u}_{1})-{\gamma }_{2}{S}_{V}-{b}_{0}{u}_{3}{S}_{V}]+{\lambda }_{6}[K{S}_{V}{I}_{H}(1-{u}_{1})\\  &  & -\,({\gamma }_{2}+\eta ){E}_{V}-{b}_{0}{u}_{3}{E}_{V}]+{\lambda }_{7}[\eta {E}_{V}-{\gamma }_{2}{I}_{V}-{b}_{0}{u}_{3}{I}_{V}].\end{array}$$

We use Pontryagin’s Maximum Principle^28^ to obtain the optimal solution of the control system (2). Since ${u}_{1}^{\ast },{u}_{2}^{\ast }$ and ${u}_{3}^{\ast }$ are solutions to the control problem (2), there exist adjoint variables *λ*_*i*_ (*i* = 1, 2, 3, 4, 5, 6, 7) satisfying the following conditions:

7 $$\begin{array}{rcl}\frac{dx}{dt} & = & \frac{\partial H(t,{u}_{1}^{\ast },{u}_{2}^{\ast },{u}_{3}^{\ast },{\lambda }_{1},{\lambda }_{2},{\lambda }_{3},{\lambda }_{4},{\lambda }_{5},{\lambda }_{6},{\lambda }_{7})}{\partial \lambda },\\ 0 & = & \frac{\partial H(t,{u}_{1}^{\ast },{u}_{2}^{\ast },{u}_{3}^{\ast },{\lambda }_{1},{\lambda }_{2},{\lambda }_{3},{\lambda }_{4},{\lambda }_{5},{\lambda }_{6},{\lambda }_{7})}{\partial \lambda },\\ \frac{d\lambda }{dt} & = & \frac{\partial H(t,{u}_{1}^{\ast },{u}_{2}^{\ast },{u}_{3}^{\ast },{\lambda }_{1},{\lambda }_{2},{\lambda }_{3},{\lambda }_{4},{\lambda }_{5},{\lambda }_{6},{\lambda }_{7})}{\partial x}.\end{array}$$

**Theorem 2.** *For the optimal-control measures* ${u}_{1}^{\ast },{u}_{2}^{\ast },{u}_{3}^{\ast }$ *and the state solutions* ${S}_{H}^{\ast },{E}_{H}^{\ast },{A}_{H}^{\ast },{I}_{H}^{\ast },{S}_{V}^{\ast },{E}_{V}^{\ast },{I}_{V}^{\ast }$ of system (2), there exist adjoint variables *λ*_*i*_ (*i* = 1, 2, 3, 4, 5, 6, 7) such that

8 $$\begin{array}{ccc}{\lambda {\rm{{\prime} }}}_{1} & = & -{\lambda }_{1}c+{\lambda }_{1}{\gamma }_{1}+({\lambda }_{1}-{\lambda }_{2}){\beta }_{1}\psi {I}_{V}(1-{u}_{1})+({\lambda }_{1}-{\lambda }_{2}){\beta }_{2}\theta \alpha {I}_{V}(1-{u}_{1})\\ {\lambda {\rm{{\prime} }}}_{2} & = & -{L}_{1}-{\lambda }_{1}c-{\lambda }_{4}m\omega +{\lambda }_{2}({\gamma }_{1}+m)-{\lambda }_{3}m(1-\omega )\\ {\lambda {\rm{{\prime} }}}_{3} & = & -{L}_{2}-{\lambda }_{1}c+{\lambda }_{3}{\gamma }_{1}\\ {\lambda {\rm{{\prime} }}}_{4} & = & -{L}_{3}-{\lambda }_{1}c+({\lambda }_{5}-{\lambda }_{6})k{S}_{V}(1-{u}_{1})+{\lambda }_{4}{u}_{2}+{\lambda }_{4}({\gamma }_{1}+\mu )\\ {\lambda {\rm{{\prime} }}}_{5} & = & -{L}_{4}-{\lambda }_{5}{\Lambda }_{V}(1-{u}_{3})+({\lambda }_{5}-{\lambda }_{6})k{I}_{H}(1-{u}_{1})+{\lambda }_{5}{b}_{0}{u}_{3}\\ {\lambda {\rm{{\prime} }}}_{6} & = & -{L}_{4}-{\lambda }_{5}{\Lambda }_{V}(1-{u}_{3})+{\lambda }_{6}({\gamma }_{2}+\eta )+{\lambda }_{6}{b}_{0}{u}_{3}-{\lambda }_{7}\eta \\ {\lambda {\rm{{\prime} }}}_{7} & = & -{L}_{4}+({\lambda }_{1}-{\lambda }_{2}){\beta }_{1}\psi {S}_{H}(1-{u}_{1})+({\lambda }_{1}-{\lambda }_{2}){\beta }_{2}\theta \alpha {S}_{H}(1-{u}_{1})\\  &  & -{\lambda }_{5}{\Lambda }_{V}(1-{u}_{3})+{\lambda }_{7}({\gamma }_{2}+{b}_{0}{u}_{3}),\end{array}$$

with the transversally conditions

$${\lambda }_{1}({T}_{f})={\lambda }_{2}({T}_{f})={\lambda }_{3}({T}_{f})={\lambda }_{4}({T}_{f})={\lambda }_{5}({T}_{f})={\lambda }_{6}({T}_{f})={\lambda }_{7}({T}_{f})=0.$$

Furthermore, the controls ${u}_{1}^{\ast },{u}_{2}^{\ast },{u}_{3}^{\ast }$ are given by

9 $$\begin{array}{rcl}{u}_{1}^{\ast } & = & \max \left\{\min \left\{1,\frac{({\lambda }_{2}-{\lambda }_{1})[{\beta }_{1}\psi +{\beta }_{2}\theta \alpha ]{S}_{H}^{\ast }{I}_{V}^{\ast }+({\lambda }_{6}-{\lambda }_{5})K{S}_{V}^{\ast }{I}_{V}^{\ast }}{{B}_{1}}\right\},0\right\},\\ {u}_{2}^{\ast } & = & \max \left\{\min \left\{1,\frac{{\lambda }_{4}{I}_{H}^{\ast }}{{B}_{2}}\right\},0\right\},\\ {u}_{3}^{\ast } & = & \max \left\{\min \left\{1,\frac{{\lambda }_{5}({\varLambda }_{V}{N}_{V}^{\ast }+{b}_{0}{S}_{V}^{\ast })+{b}_{0}({\lambda }_{6}{E}_{V}^{\ast }+{\lambda }_{7}{I}_{V}^{\ast })}{{B}_{3}}\right\},0\right\}.\end{array}$$

*Proof*. To determine the required adjoint system (8) and the transversality conditions mentioned in (9), we utilize the Hamiltonian in (6). By applying the third condition of (7), we get (8). Applying the second condition of (7), we get (9).

## Numerical Results

Unless mentioned otherwise, we use the fourth-order Runge–Kutta method over a timescale of 100 days. The input parameters for our simulations are *L*_1_ = 0.01, *L*_2_ = 0.002, *L*_3_ = 0.0020, *L*_4_ = 0.003, *B*_1_ = 0.10, *B*_2_ = *B*_3_ = 10, *c* = 0.001241, *b*_0_ = 0.21; all other parameter values are shown in Table 1.

**Table 1:** Parameter interpretations and their sample values used in numerical simulations.

Parameter	Interpretation	Values	References
*β*_1_	transmission probability during maturation	0.0016600 day^−1^	^29^
*β*_2_	transmission probability of nematode through oviposition	0.0004000 day^−1^	^29^
*ψ*	contacts averagely made during maturation per day	0.2000000 day^−1^	^30^
*γ*_1_	natural death rate of host pine trees	0.0000301 day^−1^	^31^
*γ*_2_	natural death rate of vector beetles	0.0011764 day^−1^	^32^
*θ*	contacts averagely made during oviposition per day	0.0023000 day^−1^	assumed
*m*	progression rate of pine trees from *E*_*H*_ to *I*_*H*_	0.0133000 day^−1^	assumed
Λ_*V*_	recruitment rate of susceptible vector	0.0132652 day^−1^	assumed
*K*	the rate at which the adult beetles carry PWN when they escape from dead trees	0.00305 day^−1^	^33^
*η*	progression rate of vectors from *E*_*V*_ to *I*_*V*_	0.0100000 day^−1^	assumed
*α*	probability that host susceptible cease oleoresin exudation without infected by the nematode	0.0032000 day^−1^	assumed
Λ_*H*_	recruitment rate of host trees	0.0020210 day^−1^	assumed
*ω*	rate of symptomatic cases	0.1000000	assumed
*μ*	transfer rate from *E*_*V*_ to *I*_*V*_	0.0022000 day^−1^	assumed

### Elimination of infected trees (*u*_2_) and spraying of insecticides (*u*_3_)

**Figure 3:**
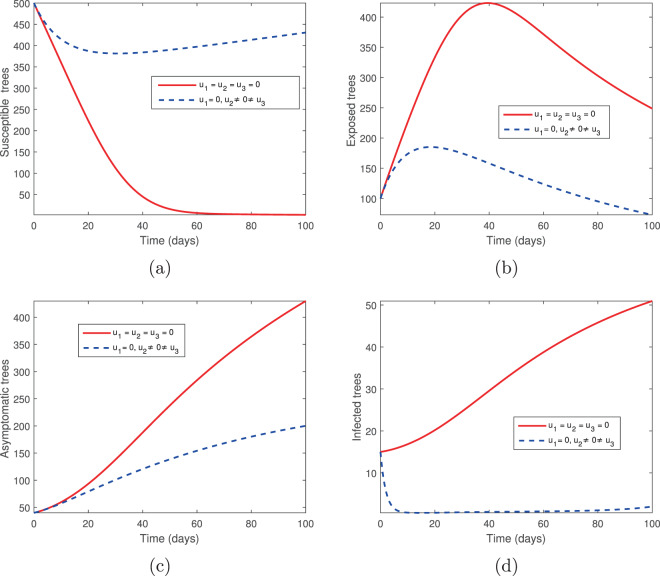
The behaviour of the pine-tree population for the controls *u*_2_ and *u*_3_; (**a**) Susceptible pine trees, (**b**) Exposed pine trees, (**c**) Asymptomatic pine trees, (**d**) Infected pine trees.

**Figure 4:**
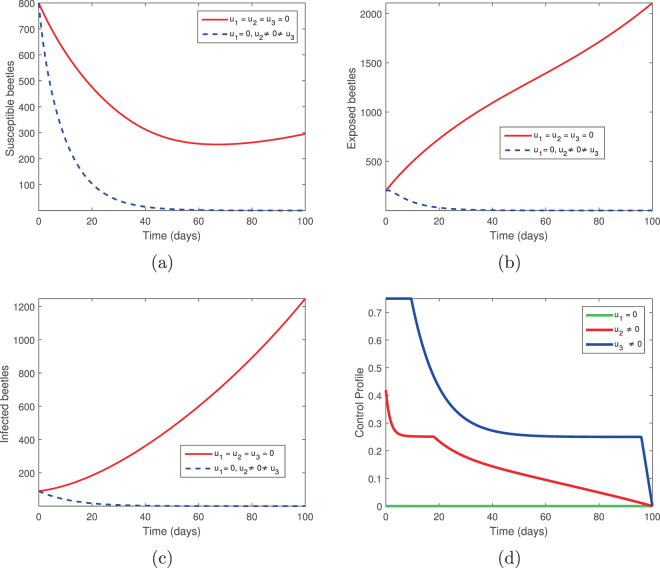
The behaviour of the vector (beetles) population for the controls *u*_2_ and *u*_3_; (**a**) Susceptible beetles, (**b**) Exposed beetles, (**c**) Infected beetles, (**d**) Control profile.

We considered two controls: the elimination of infected trees (*u*_2_) and the spraying of insecticides (*u*_3_) in the absence of tree injection and vaccination. Figures 3 and 4 show the outcomes in both the absence and presence of control. Figure 3 shows the dynamics of the pine-tree population, while Fig. 4 shows the dynamics of the vector population. With these controls, we see a rapid increase in the population of susceptible trees (Fig. 3(a)) and eventual elimination of exposed and infected trees (Fig. 3(b,d)), with only the asymptomatic carriers remaining in the infected classes (Fig. 3(c)). The vector population is eventually depleted (Fig. 4(a–c)) in the presence of these two controls. The two control profiles *u*_2_ and *u*_3_ are bounded up to 0.4 and 0.8 (Fig. 4(d)). Biologically, *u*_2_ is the additional elimination rate of only infected trees, while *u*_3_ acts to simultaneously increase the removal rate of all vectors, while also decreasing the birth rate. Since all interventions range between 0 (no control) and 1 (complete control), this suggests that our objective can be achieved with only partial controls. Hence if infected trees are removed 2.5 days or later after infection or if insecticides/larvacides are up to 80% effective, the infection can be controlled.

### Tree injection (*u*_1_) and spraying of insecticides (*u*_3_)

**Figure 5:**
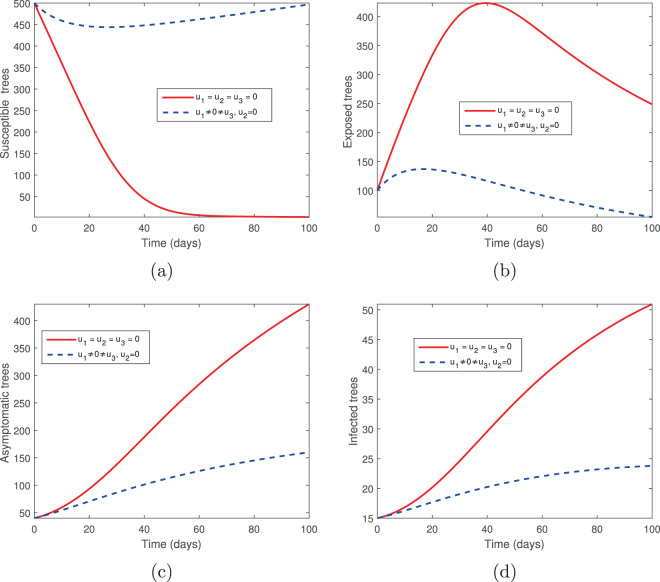
The behaviour of the pine-tree population for the controls *u*_1_ and *u*_3_; (**a**) Susceptible pine trees, (**b**) Exposed pine trees, (**c**) Asymptomatic pine trees, (**d**) Infected pine trees.

**Figure 6:**
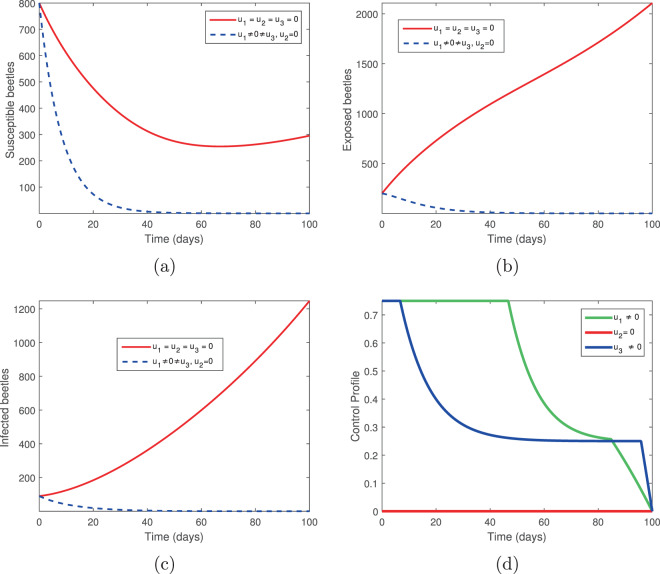
The behaviour of the vector (beetles) population for the controls *u*_1_ and *u*_3_; (**a**) Susceptible beetles, (**b**) Exposed beetles, (**c**) Infected beetles, (**d**) Control profile.

We next examine the combination of tree injection (*u*_1_) and insecticide spraying (*u*_3_). The results are shown in Figs. 5 and 6. With these two controls, there is a significant increase in the population of susceptible and exposed pine trees, while the population of asymptomatic carriers and infected pine trees are reduced but not eliminated (Fig. 5). This suggests that the elimination of infected pine trees has a significant impact on the disease. Note that the vector population is eliminated using these controls (Fig. 6).

### Tree injection (*u*_1_) and elimination of infected trees (*u*_2_)

**Figure 7:**
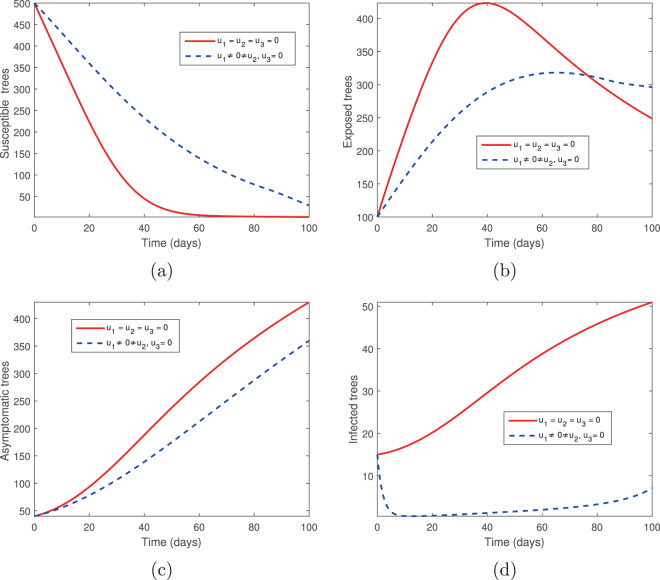
The behaviour of the pine-tree population for the controls *u*_1_ and *u*_2_; (**a**) Susceptible pine trees, (**b**) Exposed pine trees, (**c**) Asymptomatic pine trees, (**d**) Infected pine trees.

**Figure 8:**
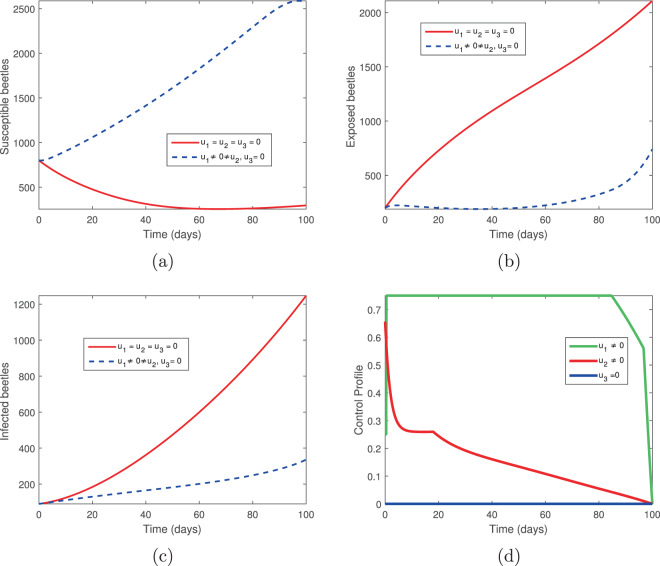
The behaviour of the vector (beetles) population for the controls *u*_1_ and *u*_2_; (**a**) Susceptible beetles, (**b**) Exposed beetles, (**c**) Infected beetles, (**d**) Control profile.

Considering *u*_1_ and *u*_2_ in combination, Figs. 7 and 8 illustrate that, without insecticide spraying, the control (minimization and/or elimination) of infection in the pine trees is not possible. While the population of susceptible pine trees has a slower decline with these control (Fig. 7(a)), the infection eventually takes over. Likewise, although the susceptible beetle population is recovered using these controls, the infection nevertheless eventually dominates (Fig. 8). It follows that, without insecticide spraying, the control of infection is not possible.

### Complete control

**Figure 9:**
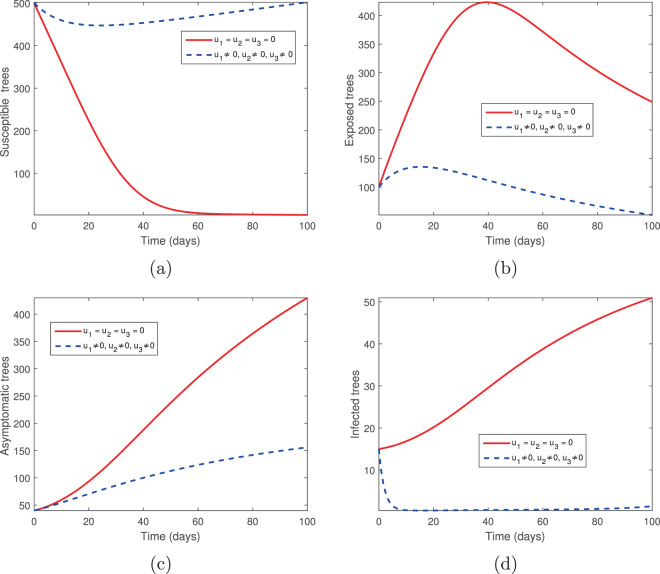
The behaviour of the pine-tree population for the controls *u*_1_, *u*_2_ and *u*_3_; (**a**) Susceptible pine trees, (**b**) Exposed pine trees, (**c**) Asymptomatic pine trees, (**d**) Infected pine trees.

**Figure 10:**
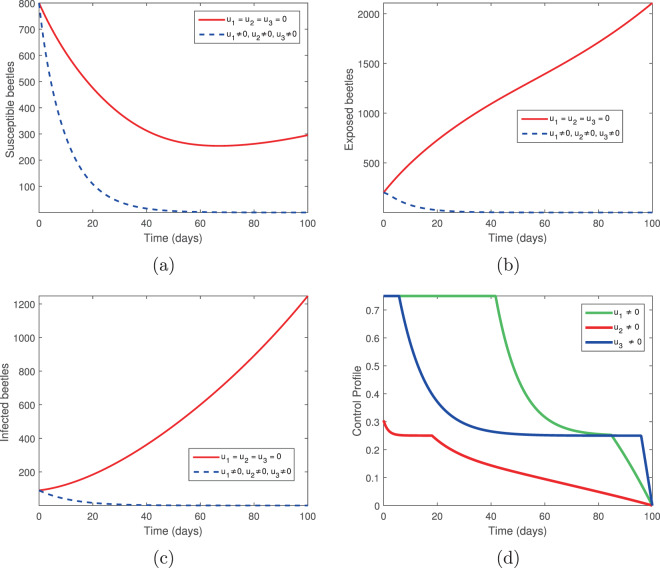
The behaviour of the vector (beetles) population for the controls *u*_1_, *u*_2_ and *u*_3_; (**a**) Susceptible beetles, (**b**) Exposed beetles, (**c**) Infected beetles, (**d**) Control profile.

We now apply all three controls in order to determine the ideal outcome (Figs. 9 and 10). Comparing Fig. 9 to Fig. 3, we see that susceptible pine trees recover faster and the disease is eliminated quicker, except for asymptomatic carriers. We thus see that the most effective strategy is to apply all three controls, although similar results can be achieved by applying only two controls: elimination of the infected pine trees (*u*_2_) and the spraying of insecticides (*u*_3_).

### Temporal variation of control profiles

**Figure 11:**
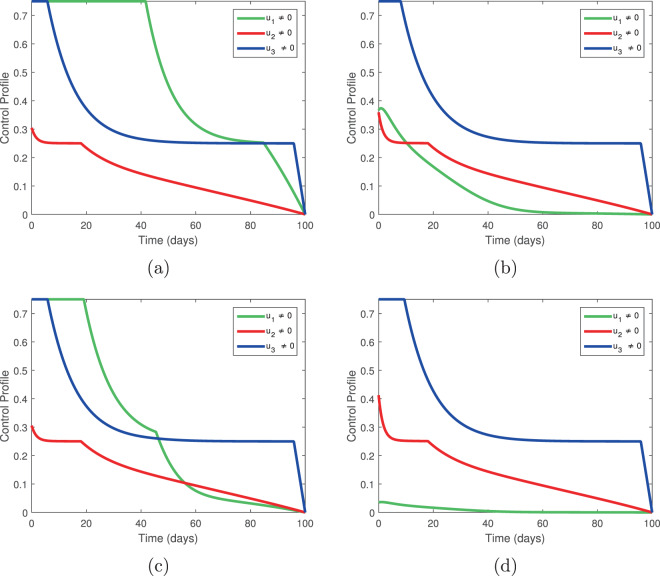
Temporal variation of the control profile for *L*_1_ = 0.01, *L*_2_ = 0.002, *L*_3_ = 0.0020, *L*_4_ = 0.003, *B*_2_ = *B*_3_ = 10; (**a**) *B*_1_ = 0.10, (**b**) *B*_1_ = 1, (**c**) *B*_1_ = 10, (**d**) *B*_1_ = 100.

**Figure 12:**
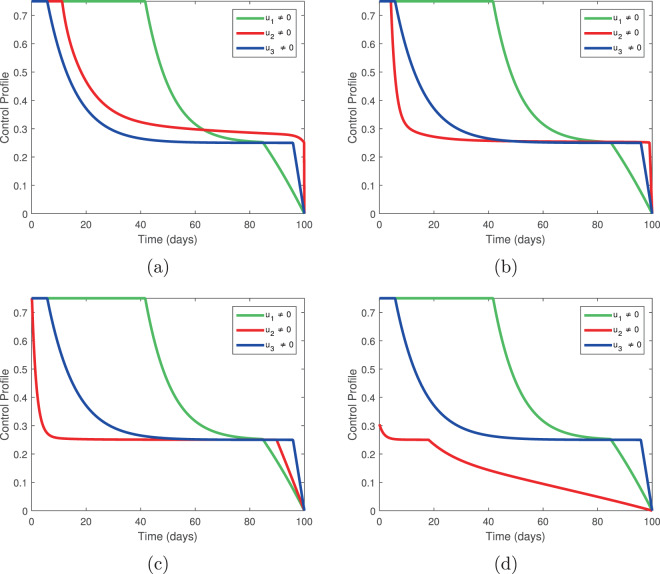
Temporal variation of the control profile for *L*_1_ = 0.01, *L*_2_ = 0.002, *L*_3_ = 0.0020, *L*_4_ = 0.003, *B*_1_ = 0.1, *B*_3_ = 10; (**a**) *B*_2_ = 0.010, (**b**) *B*_2_ = 0.10, (**c**) *B*_2_ = 1, (**d**) *B*_2_ = 10.

**Figure 13:**
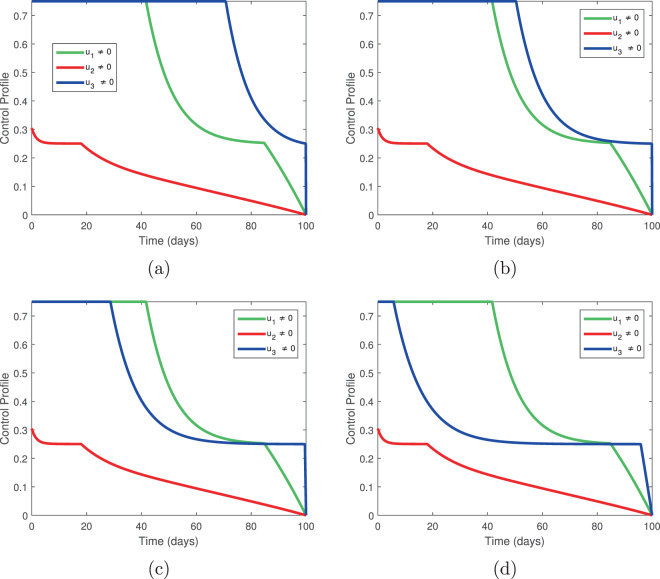
Temporal variation of the control profile for *L*_1_ = 0.01, *L*_2_ = 0.002, *L*_3_ = 0.0020, *L*_4_ = 0.003, *B*_1_ = 0.1, *B*_2_ = 10; (**a**) *B*_3_ = 0.010, (**b**) *B*_3_ = 0.1, (**c**) *B*_3_ = 1, (**d**) *B*_3_ = 10.

Next, we investigate the control profiles and their relationships to the weight constants. In Fig. 11, we fix the weight constants *L*_1_ = 0.01, *L*_2_ = 0.002, *L*_3_ = 0.0020, *L*_4_ = 0.003, *B*_2_ = *B*_3_ = 10 and allow *B*_1_ to vary. In Fig. 12, we fix the weight constants *L*_1_ = 0.01, *L*_2_ = 0.002, *L*_3_ = 0.0020, *L*_4_ = 0.003, *B*_1_ = 0.1, *B*_3_ = 10 and allow *B*_2_ to vary. In Fig. 13, we fix *L*_1_ = 0.01, *L*_2_ = 0.002, *L*_3_ = 0.0020, *L*_4_ = 0.003, *B*_1_ = 0.1, *B*_2_ = 10 and allow *B*_3_ to vary. These variations represent fluctuating costs of implementing our controls.

From Fig. 11, we see that, as the cost of *u*_1_ increases, the control profile is dominated by *u*_3_. That is, if tree injection becomes prohibitively expensive, the procedure can be replaced by increased insecticide spraying.

Figure 12 shows little variation in the control profiles as the cost *B*_2_ increases unless the cost is prohibitive. This suggests that the control *u*_2_ is worth implementing, even at high cost. The combination of *u*_1_ and *u*_3_ alone does not eliminate infection, so it follows that elimination of infected trees is essential to disease control. This may hinder disease eradication if the costs of elimination become prohibitively expensive.

Figure 13 shows that if the cost of insecticide spraying increases, the control profile is dominated by tree injection. Interestingly, while the combination of *u*_2_ and *u*_3_ produced superior results to the combination of *u*_1_ and *u*_2_, the latter combination can still produce effective results if supplemented by a small amount of insecticide spraying.

## Discussion

We developed a mathematical model to examine the effect of asymptomatic carriers of Pine Wilt Disease (PWD) on the long-term course of disease. We showed that the disease-free equilibrium was globally asymptotically stable and that the endemic equilibrium was globally asymptotically stable under some conditions. A sensitivity analysis identified key parameters: natural death rates in trees and beetles; birth rates in both trees and beetles; and transmission rates from trees to beetles.

We applied several controls to our system: tree injection, insecticide spraying and elimination of infected trees. These were chosen in conjunction with the most sensitive parameters except for the natural birth and death rates of trees, since our ultimate goal is the preservation of trees. We showed that the disease can be eliminated using suitable controls, except for the asymptomatic carriers. By including this class, our model showed that the disease may remain endemic, requiring permanent control, even in the best-case scenario.

Examining the controls in detail, we found that elimination of infected trees is critical, especially when used in conjunction with insecticide spraying. If the cost of insecticide spraying becomes prohibitive, it can be partially replaced by tree injection. However, if the costs of elimination of infected trees becomes prohibitive, there is no simple replacement, which may result in runaway costs.

It follows that we can control the disease using suitable interventions, but it will not be eliminated due to the presence asymptomatic carriers. The presence of infection in these carriers suggests that infection can restart in nearby healthy trees. It follows that our control measures must be undertaken continually unless such asymptomatic carriers can be identified and removed. This has long-term implications for disease management and economic investment.

## Supplementary information

Supplementary Information.
