# Supplementary material for: Modelling the dynamics of Pine Wilt Disease with asymptomatic carriers and optimal control
Source: Sci Rep. 2020 Jul 10;10:11412. doi: 10.1038/s41598-020-67090-7 (PMC7351782; doi:10.1038/s41598-020-67090-7)
Supplement: Supplementary file 1 — Supplementary Information. [file 41598_2020_67090_MOESM1_ESM.pdf]

Modelling the dynamics of Pine Wilt Disease with asymptomatic carriers and optimal control: Supplementary Material

M.A. Khan[1,2], L. Ahmed[3], Prashanta Kumar Mandal[4], Robert Smith?[5], Mainul Haque[6,*]

[1] Informetrics Research Group, Ton Duc Thang University, Ho Chi Minh City, Vietnam

[2] Faculty of Mathematics and Statistics, Ton Duc Thang University, Ho Chi Minh City, Vietnam

[3] Department of Mathematics, City University of Science and Information Technology, Peshwar, Pakistan

[4] Department of Mathematics, Visva-Bharati University, Santiniketan-731 235, W.B., India

[5,*] Department of Mathematics and Faculty of Medicine, The University of Ottawa, Ottawa, ON, K1N 6N5, Canada

[6,*] Department of Mathematics and Physics University of Portsmouth, Portsmouth PO1 2UP, UK

Stability analysis of the model

There are two biologically meaningful equilibria of the PWD model: the disease-free equilibrium (DFE) and the endemic equilibrium (EE). The former is given by

$$E_0 = \left(\frac{\Lambda_H}{\gamma_1}, 0, 0, 0, \frac{\Lambda_V}{\gamma_2}, 0, 0\right).$$

Using the next-generation method [1], we find the repoduction number is

$$\mathcal{R}_0 = \rho(FV^{-1}) = \sqrt{\frac{mK\omega\varLambda_V\varLambda_H\eta(\beta_2\theta\alpha + \beta_1\psi)}{\gamma_1\gamma_2^2(m+\gamma_1)(\mu+\gamma_1)(\eta+\gamma_2)}}.$$

Note that this value is a threshold for disease emergence, not necessarily the average number of secondary infections [2].

Global stability of the DFE

Here, we prove the global stability of the DFE E_0 using the approach from Castillo-Chavez and Huang [3]. We rewrite our model as follows:

$$\begin{aligned} \frac{dY}{dt} &= F(Y,Z), \\ \frac{dZ}{dt} &= M(Y,Z), \quad M(Y,0)=0, \end{aligned} \tag{1}$$

where $Y = (S_H, S_V) \in \mathbb{R}^2_+$, represent the number of uninfected compartments and $Z = (E_H,\ A_H,\ I_H,\ E_V,\ I_V) \in \mathbb{R}^5_+$, represent the infected tree and vector classes. The DFE is $(Y^0,\ 0)$, where $Y^0 = \left(\frac{\Lambda_H}{\gamma_1}, \frac{\Lambda_V}{\gamma_2}\right)$. For global stability of the DFE, the following two conditions need to be satisfied:

$$\begin{aligned} &(C_1) \text{ For } \frac{dY}{dt} = F(Y,0) = 0,\ Y^0 \text{ is globally asymptotically stable} \\ &(C_2)\ M(Y,Z) = AZ - \widehat{G}(Y,Z),\ \text{where } \widehat{G}(Y,Z) \geq 0,\ \text{for } (Y,Z) \in \Omega, \end{aligned}$$

and where $A = D_z M(Y^0, 0)$ is an M-matrix and Ω is the biological feasible region.

Lemma 1. *If $\mathcal{R}_0 < 1$, then the fixed point denoted by $(Y^0, 0)$ of system (1) is globally asymptotically stable if (C_1) and (C_2) are satisfied.*

For the proof of Lemma 1 where the conditions are proved in general, see [3], but in particular case, these conditions are proven in below theorem.

Theorem 1. *If $\mathcal{R}_0 < 1$ and assumptions (C_1) and (C_2) are satisfied, then the DFE E_0 is gobally asymptotically stable.*

Proof. Let

$$F(X,0) = \begin{pmatrix} \Lambda_H - \gamma_1 S_H^0 \\ \Lambda_V - \gamma_2 S_V^0 \end{pmatrix}.$$

As $t \to \infty$, and $Y \to Y^0$, $Y = Y^0 = (S_H^0, S_V^0)$ is globally asymptotically stable. To ensure condition (C_2), let

$$A = \begin{pmatrix} -\tau_1 & 0 & 0 & 0 & \tau_4 S_H^0 \\ m(1-\omega) & -\gamma_1 & 0 & 0 & 0 \\ m\omega & 0 & -\tau_2 & 0 & 0 \\ 0 & 0 & K S_V^0 & -\tau_3 & 0 \\ 0 & 0 & 0 & \eta & -\gamma_2 \end{pmatrix},$$

$$Z = \begin{pmatrix} E_H \\ A_H \\ I_H \\ E_V \\ I_V \end{pmatrix} \text{ and } \widehat{G}(Y,Z) = \begin{pmatrix} \tau_4 S_H^0 (1 - \frac{S_H}{S_H^0}) \\ 0 \\ 0 \\ K S_V^0 I_H (1 - \frac{S_V}{S_V^0}) \\ 0 \end{pmatrix}.$$

where $\tau_4 = \alpha\beta_2\theta + \beta_1\psi$. Then $M(Y,Z)$ can be written as $M(Y,Z) = AZ - \widehat{G}(Y,Z)$. Clearly, $\widehat{G}(Y,Z) \geq 0$ and A is an M-matrix with negative diagonals. Hence conditions (C_1) and (C_2) are fulfilled. Thus, by Lemma 1, E_0 is globally asymptotically stable. □

Note that the global stability of the DFE implies local stability and also that no backward bifurcation is possible.

Global stability of the endemic equilibrium

Next, we will prove global stability of EE^* [4, 5, 6]. At the EE, the PWD model at steady state satisfies

$$\begin{aligned}
\Lambda_H &= \tau_4 S_H^* I_V^* + \gamma_1 S_H^*, & \tau_1 E_H^* &= \tau_4 S_H^* I_V^* \\
m(1-\omega)E_H^* &= \gamma_1 A_H^* & mE_H^* &= \tau_2 I_H^*, \\
\frac{\tau_1\tau_2}{m} I_H^* &= \tau_4 S_H^* I_V^* & \Lambda_V &= K S_V^* I_H^* + \gamma_2 S_V^* \\
K S_V^* I_H^* &= \tau_3 E_V^*, & \eta E_V^* &= \gamma_2 I_V^*, \\
K S_V^* I_H^* &= \frac{\tau_3 \gamma_2 I_V^*}{\eta}
\end{aligned}$$

Theorem 2. *If $\mathcal{R}_0 > 1$ and*

$$\left(7 - \frac{S_H^*}{S_H} - \frac{S_H I_V E_H^*}{S_H^* I_V^* E_H} - \frac{A_H}{A_H^*} - \frac{E_H A_H^*}{A_H E_H^*} - \frac{E_H I_H^*}{I_H E_H^*} + \frac{E_H}{E_H^*} - \frac{S_V^*}{S_V} - \frac{S_V I_H E_V^*}{S_V^* E_V I_H^*} - \frac{E_V I_V^*}{I_V E_V^*}\right) \leq 0,$$

then the endemic equilibrium EE^* is globally asymptotically stable

Proof. Consider the Lyapunov function

$$\begin{aligned}
L &= K S_V^* I_H^* \Bigg[\int_{S_H^*}^{S_H} \left(1 - \frac{S_H^*}{x}\right) dx + \int_{E_H^*}^{E_H} \left(1 - \frac{E_H^*}{x}\right) dx + \frac{\tau_4 S_H^* I_V^*}{m(1-\omega)E_H^*} \int_{A_H^*}^{A_H} \left(1 - \frac{A_H^*}{x}\right) dx \\
&\quad + \frac{\tau_4 S_H^* I_V^*}{m\omega E_H^*} \int_{I_H^*}^{I_H} \left(1 - \frac{I_H^*}{x}\right) dx \Bigg] + \tau_4 S_H^* I_V^* \Bigg[\int_{S_V^*}^{S_V} \left(1 - \frac{S_V^*}{x}\right) dx + \int_{E_V^*}^{E_V} \left(1 - \frac{E_V^*}{x}\right) dx \\
&\quad + \frac{K S_V^* I_H^*}{\eta E_V^*} \int_{I_V^*}^{I_V} \left(1 - \frac{I_V^*}{x}\right) dx \Bigg].
\end{aligned}$$

We have

$$\begin{aligned}
L' &= K S_V^* I_H^* \Bigg[\left(1 - \frac{S_H^*}{S_H}\right) S_H' + \left(1 - \frac{E_H^*}{E_H}\right) E_H' + \frac{\tau_4 S_H^* I_V^*}{m(1-\omega)E_H^*} \left(1 - \frac{A_H^*}{A_H}\right) A_H' \\
&\quad + \frac{\tau_4 S_H^* I_V^*}{m\omega E_H^*} \left(1 - \frac{I_H^*}{I_H}\right) I_H' \Bigg] + \tau_4 S_H^* I_V^* \Bigg[\left(1 - \frac{S_V^*}{S_V}\right) S_V' + \left(1 - \frac{E_V^*}{E_V}\right) E_V' + \frac{K S_V^* I_H^*}{\eta E_V^*} \left(1 - \frac{I_V^*}{I_V}\right) I_V' \Bigg].
\end{aligned}$$

Simplifying, we get the following results:

$$
\begin{aligned}
\left(1-\frac{S_H^*}{S_H}\right)S_H' &= \left(1-\frac{S_H^*}{S_H}\right)[\Lambda_H-\tau_4 S_H I_V-\gamma_1 S_H]\\
&= \left(1-\frac{S_H^*}{S_H}\right)[\tau_4 S_H^* I_V^*+\gamma_1 S_H^*-\tau_4 S_H I_V-\gamma_1 S_H]\\
&= \gamma_1 S_H^*\left(2-\frac{S_H}{S_H^*}-\frac{S_H^*}{S_H}\right)+\left(1-\frac{S_H^*}{S_H}\right)[\tau_4 S_H^* I_V^*-\tau_4 S_H I_V]\\
&= \gamma_1 S_H^*\left(2-\frac{S_H}{S_H^*}-\frac{S_H^*}{S_H}\right)+\tau_4 S_H^* I_V^*\left(1-\frac{S_H^*}{S_H}-\frac{S_H I_V}{S_H^* I_V^*}+\frac{I_V}{I_V^*}\right),\\
\left(1-\frac{E_H^*}{E_H}\right)E_H' &= \left(1-\frac{E_H^*}{E_H}\right)[\tau_4 S_H I_V-\tau_1 E_H]\\
&= \left(1-\frac{E_H^*}{E_H}\right)\left[\tau_4 S_H I_V-\frac{\tau_4 S_H^* I_V^*}{E_H^*}E_H\right]\\
&= \tau_4 S_H^* I_V^*\left(1-\frac{E_H}{E_H^*}-\frac{S_H I_V E_H^*}{S_H^* I_V^* E_H}+\frac{S_H I_V}{S_H^* I_V^*}\right),\\
\frac{\tau_4 S_H^* I_V^*}{m(1-\omega)E_H^*}\left(1-\frac{A_H^*}{A_H}\right)A_H' &= \frac{\tau_4 S_H^* I_V^*}{m(1-\omega)E_H^*}\left(1-\frac{A_H^*}{A_H}\right)[m(1-\omega)E_H-\gamma_1 A_H]\\
&= \frac{\tau_4 S_H^* I_V^*}{E_H^*}\left(1-\frac{A_H^*}{A_H}\right)\left[E_H-\frac{E_H^*}{A_H^*}A_H\right]\\
&= \tau_4 S_H^* I_V^*\left(1-\frac{A_H}{A_H^*}-\frac{E_H A_H^*}{E_H^* A_H}+\frac{E_H}{E_H^*}\right),\\
\frac{\tau_4 S_H^* I_V^*}{m\omega E_H^*}\left(1-\frac{I_H^*}{I_H}\right)I_H' &= \frac{\tau_4 S_H^* I_V^*}{m\omega E_H^*}\left(1-\frac{I_H^*}{I_H}\right)[m\omega E_H-\tau_2 I_H]\\
&= \frac{\tau_4 S_H^* I_V^*}{E_H^*}\left(1-\frac{I_H^*}{I_H}\right)\left[E_H-\frac{E_H^*}{I_H^*}I_H\right],\\
&= \tau_4 S_H^* I_V^*\left(1-\frac{I_H}{I_H^*}-\frac{E_H I_H^*}{I_H E_H^*}+\frac{E_H}{E_H^*}\right),\\
\left(1-\frac{S_V^*}{S_V}\right)S_V' &= \left(1-\frac{S_V^*}{S_V}\right)[\Lambda_V-KS_V I_H-\gamma_2 S_V]\\
&= \left(1-\frac{S_H^*}{S_H}\right)[KS_V^* I_H^*+\gamma_2 S_V^*-KS_V-\gamma_2 S_V]\\
&= \gamma_2 S_V^*\left(2-\frac{S_V}{S_V^*}-\frac{S_V^*}{S_V}\right)+KS_V^* I_H^*\left(1-\frac{S_V I_H}{S_V^* I_H^*}-\frac{S_V^*}{S_V}+\frac{I_H}{I_H^*}\right),\\
\left(1-\frac{E_V^*}{E_V}\right)E_V' &= \left(1-\frac{E_V^*}{E_V}\right)[KS_V I_H-\tau_3 E_V]\\
&= \left(1-\frac{E_V^*}{E_V}\right)\left[KS_V I_H-\frac{KS_V^* I_H^* E_V}{E_V^*}\right]\\
&= KS_V^* I_H^*\left(1-\frac{E_V}{E_V^*}-\frac{S_V I_H E_V^*}{E_V S_V^* I_H^*}+\frac{S_V I_H}{S_V^* I_H^*}\right),
\end{aligned}
$$

and

$$\left(1-\frac{I_V^*}{I_V}\right)\frac{KS_V^*I_H^*}{\eta E_V^*}I_V' = \frac{KS_V^*I_H^*}{\eta E_V^*}\left(1-\frac{I_V^*}{I_V}\right)[\eta E_V - \gamma_2 I_V]$$
$$= \frac{KS_V^*I_H^*}{E_V^*}\left(1-\frac{I_V^*}{I_V}\right)[E_V - \frac{E_V^*}{I_V^*}I_V]$$
$$= KS_V^*I_H^*\left(1-\frac{I_V}{I_V^*}-\frac{E_VI_V^*}{I_VE_V^*}+\frac{E_V}{E_V^*}\right).$$

It follows from the above equations that

$$\begin{aligned} L'(t) &= \tau_4 KS_H^*I_V^*S_V^*I_H^*\Big[7-\frac{S_H^*}{S_H}-\frac{S_HI_VE_H^*}{S_H^*I_V^*E_H}-\frac{A_H}{A_H^*}-\frac{E_HA_H^*}{A_HE_H^*}-\frac{E_HI_H^*}{I_HE_H^*}+\frac{E_H}{E_H^*} \\ &-\frac{S_V^*}{S_V}-\frac{S_VI_HE_V^*}{S_V^*E_VI_H^*}-\frac{E_VI_V^*}{I_VE_V^*}\Big]+\gamma_1KS_H^*S_V^*I_H^*\left(2-\frac{S_H}{S_H^*}-\frac{S_H^*}{S_H}\right) \\ &+\tau_4\gamma_2S_H^*I_V^*S_V^*\left(2-\frac{S_V}{S_V^*}-\frac{S_V^*}{S_V}\right). \end{aligned}$$

in which

$$\left(2-\frac{S_H}{S_H^*}-\frac{S_H^*}{S_H}\right)\leq 0,$$
$$\left(2-\frac{S_V}{S_V^*}-\frac{S_V^*}{S_V}\right)\leq 0,$$

and if

$$\left[7-\frac{S_H^*}{S_H}-\frac{S_HI_VE_H^*}{S_H^*I_V^*E_H}-\frac{A_H}{A_H^*}-\frac{E_HA_H^*}{A_HE_H^*}-\frac{E_HI_H^*}{I_HE_H^*}+\frac{E_H}{E_H^*}-\frac{S_V^*}{S_V}-\frac{S_VI_HE_V^*}{S_V^*E_VI_H^*}-\frac{E_VI_V^*}{I_VE_V^*}\right]\leq 0,$$

then the largest invariant subset for which $L'=0$ is EE^*. By LaSalle's Invariance Principle [7], EE^* is globally asymptotically stable whenever $\mathcal{R}_0>1$. □

References

[1] Driessche, P.V.D. & Watmough, J. Reproduction numbers and sub-threshold endemic equilibria for compartmental models of disease transmission. *Math. Biosci.* **180**, 29–48 (2002).

[2] Heffernan, J.M., Smith, R.J. & Wahl, L.M. Perspectives on basic reproduction ratio. *J. R. Soc. Inter.* **2**, 281–293 (2005).

[3] Castillo-Chavez, C., Feng, Z. & Huang, W. On the computation of R0 and its role on global stability. *Mathematical Approaches for Emerging and Re-Emerging Infection Diseases: An Introduction. The IMA Volumes in Mathematics and Its Applications.* **125**, 31–65 (2002).

[4] Gui-Quan, S., Xie, S.H., Jin, Z, *et al.* Transmission Dynamics of Cholera: Mathematical Modeling and Control Strategies. *Comm. Nonl. Sci. Num. Sim.*,**45**, 235–244 (2017).

[5] Ming-Tao, L., Zhen, J., Gui-Quan, S. & Juan, Z. Modeling direct and indirect disease transmission using multi-group mode. *J. Math. Anal. Appl.* **446**(2), 1292–1309 (2017).

[6] Guo, H., Li, M.Y. & Shuai, Z. A graph-theoretic approach to the method of global Lyapunov functions. *Proc. Am. Math. Soc.* **136**, 2793–2802 (2008).

[7] Lasalle, J.P. Stability theory for difference equations. In: Hale, J. K. (Ed.) *Studies in Ordinary Differential Equations.* Washington DC: Math. Assoc. of America (1977).
